# The influence of medical insurance on the use of basic public health services for the floating population: the mediating effect of social integration

**DOI:** 10.1186/s12939-022-01623-6

**Published:** 2022-02-03

**Authors:** Yulin Li, Dongmei Dou

**Affiliations:** School of Nursing and Health, Henan University, Kaifeng, 475004 China

**Keywords:** Medical insurance, Basic public health services, Social integration

## Abstract

**Background:**

The accessibility and fairness of the floating population’s access to basic public health services have an important impact on improving the health level of the whole population. Existing studies have shown that medical insurance and social integration have an impact on basic public health services, but there are few studies on the specific influence path between the three. Therefore, the research purpose of this paper is to explore the effects of medical insurance for urban and rural residents and basic medical insurance for urban employees on the utilization of basic public health services, and to analyze the mediating effect of social integration.

**Methods:**

The data in this paper are derived from the 2017 China Mobile Population Dynamic Monitoring Survey data, which collects information on 31 provinces (regions, cities) and Xinjiang Production and Construction Corps mobile population 169,989 people, all of whom come from China’s relatively concentrated mobile population inflow areas (NHC FPSCo. 2021. Floating Population Service Center of NHC). After deleting part of the missing data and replacing the mean value, 154,586 people were finally included in the analysis. The proportion is 90.9%. Based on the data of China’s floating population dynamic survey in 2017,we used Logistic regression method to analyze the effects of basic medical insurance for urban and rural residents, basic medical insurance for urban employees and social integration on the utilization of basic public health services. Then we used the Bootstrap method of structural equation model to analyze the mediating effect of social integration.

**Results:**

Medical insurance for urban and rural residents (β = 0.236;95%CI:1.195 ~ 1.342) has positive impact on health education, it (β = 0,190;95%CI:1.150 ~ 1.272) also has positive impact on the establishment of residents’ health records. Social integration (including political participation (β = 0.312;95%CI:1.324 ~ 1.410),activity participation (β = 0.724;95%CI:2.009 ~ 2.119), identity (β = 0.421; 95%CI:1.387 ~ 1.675))has positive impact on health education, it ((β = 0.312;95%CI:1.324 ~ 1.410), (β = 0.404;95%CI:1.463 ~ 1.534), (β = 0.282;95%CI:1.191 ~ 1.477)) also has positive impact on the establishment of residents’ health records. In addition, BMIUE (β = 0.169;95%CI:1.150 ~ 1.219) has an impact on the establishment of residents’ health records. The direct effect of medical insurance on the utilization of public health services was 0.092 (95%CI: 0.093 ~ 0.103), social integration was a partial mediator, the mediating effect was 0.127 (95%CI: 0.108 ~ 0.127), and the mediating effect size was 57.73%.

**Conclusions:**

Medical insurance can directly promote the floating population to use basic public health services, and can indirectly promote the improvement of public health service utilization level through social integration.

## Background

The floating population is defined as individuals whose registered permanent residence is their original residence, and they live and work in a current residence that is not their registered permanent residence [1]. The report of the 19th National Congress of the Communist Party of China stressed the importance of implementing the Health China Strategy and accelerating the equalization of public health services. The implementation of the national basic public health service project is an important part of promoting the gradual equalization of basic public health services. According to the 7th National Census Bulletin issued by the National Bureau of Statistics, China’s floating population has reached 376 million [1], and is expected to grow in the next few years. Because of the high mobility of the floating population, studying the utilization of public health services for the floating population can not only improve the fairness and accessibility of the floating population’s access to public health services, but also be of great significance for improving the health level of the whole population [2, 3]. Now there are studies showing that participation in medical insurance and better social integration will promote the use of public health services by the floating population [4–6], and the comprehensive impact of participation in medical insurance on social integration is gradually increasing [7], but the relationship between medical insurance, social integration, and the use of basic public health services is less studied by scholars. Therefore, this paper takes as an example participating in medical insurance for urban and rural residents and basic medical insurance for urban employees (BMIUE), and attempts to explore the relationship between the three,so as to provide a realistic basis for improving the level of utilization of basic public health services for the floating population.

## Methods

### Data source

The China Migrants Dynamic Survey (CMDS) is an annual sample Survey of Migrants conducted by the NHC since 2009. It covers 31 provinces (autonomous regions and municipalities directly under the Central government) and areas where the floating population is concentrated in Xinjiang Production and Construction Corps, with a sample size of nearly 200,000 households per year [8]. The data in this paper are derived from the 2017 China Mobile Population Dynamic Monitoring Survey data, which collects information on 31 provinces (regions, cities) and Xinjiang Production and Construction Corps mobile population 169,989 people, all of whom come from China’s relatively concentrated mobile population inflow areas [9]. After deleting part of the missing data and replacing the mean value, 154,586 people were finally included in the analysis. The proportion is 90.9%.

### Measurements

In China’s public health service utilization project, only health education and the establishment of health files are aimed at the whole population. Dependent variables were selected as indicators to measure the utilization of public health services, including the status of receiving health education and the status of establishing health records of residents after the treatment of binary classification. The independent variable is medical insurance, including basic medical insurance for urban and rural residents and BMIUE. As a mediating variable, social integration includes political participation, activity participation [10] and identity [11]. Each is measured by whether the following behaviors occur: Offer advice to place unit/community/village or supervision unit/community/village management, ““through various methods to report the situation to the relevant government departments/put forward policy recommendations” “online comment on national affairs and social events, participate in discussion”, “actively participate in the donation, unpaid blood donation and volunteer activities such as” “to participate in the activities of the party/youth league organizations, “I like the city/place where I live”, “I would like to fit in with the locals and be part of them”, “I think the locals would like to accept me as part of them”. Control variables include age, sex, marital status, education level, nature of household registration, average monthly household expenditure in the past year, flow range and flow time.

### Statistical method

SPSS 25.0 was used for descriptive analysis, univariate analysis and multivariate logistic regression analysis. AMOS 25.0 was used to establish structural equation model and construct a standardized path test.;Bootstrapping was used to test the mediating effects. The *p*-value criteria for univariate and multivariate logistic regression analysis were *P* < 0.05; Similarly, the test criteria of structural equation model was the model fitting index, and the specific evaluation criteria were GFI,AGFI and CFI > 0.9,and RMSEA< 0.05, showing that the modle had good validity.

## Results

### Description

Among 154,586 floating population, 46,389 (30%) had established health records, and the filing rate was relatively low. A total of 112,989 people (73.10%) had received health education, with a high acceptance rate. A total of 147,121 (95.17%) were aged between 18 and 60 years old, and 7465 (4.83%) were over 60 years old. There were 79,577 males (51.48%) and 75,009 females (48.52%); 21,063 (13.63%) were unmarried, 128,038 (82.83%) were married, and 5485 (3.55%) were divorced. There were 119,675 (77.42%) in agricultural households and 22,858 (14.79%) in urban households; 26,358 students (17.05%) received education in primary schools and below, 67,181 students (43.46%) in junior middle schools, 33,785 students (21.86%) in senior high schools and technical secondary schools, and 27,262 students (17.64%) in junior college or above. 76,673 people (49.60%) spent 1000–3000 yuan per month, followed by 44,498 people (28.79%) spent 3000–5000 yuan per month. There were 74,875 (48.44%) people across provinces, 51,682 (33.43%) people across cities, and 28,029 (18.13%) people across counties within cities. 81,793 people (52.91%) had a migration time less than 8 years, followed by 43,688 people (28.26%) from 8 to 14 years. A total of 7438 people (4.81%) participated in medical insurance for urban and rural residents, and 34,411 people (22.26%) participated in medical insurance for urban workers; 21,823 (14.10%) showed good political participation, 58,574 (37.90%) showed good activity participation and 152,634 (98.70%) showed good identity.

### Regression analysis of the utilization of public health services by medical insurance and social integration

The dependent variables were whether the floating population had received health education (0 = no, 1 = yes) and whether the floating population had established health records (0 = no, 1 = yes). The independent variables were including gender, age, marital status, education level, nature of household registration, average monthly household expenditure, mobile range, mobile time, participation in medical insurance for urban and rural residents, participation in BMIUE and social integration, which were statistically significant in univariate analysis. The results of multivariate Logistic regression analysis showed that: Medical insurance for urban and rural residents (β = 0.236;95%CI:1.195 ~ 1.342) has positive impact on health education, it (β = 0,190;95%CI:1.150 ~ 1.272) also has positive impact on the establishment of residents’ health records. Social integration(including political participation (β = 0.312; 95%CI:1.324 ~ 1.410), activity participation(β = 0.724;95%CI:2.009 ~ 2.119), identity (β = 0.421; 95%CI:1.387 ~ 1.675))has positive impact on health education, it ((β = 0.312;95%CI:1.324 ~ 1.410), (β = 0.404;95%CI:1.463 ~ 1.534), (β = 0.282;95%CI:1.191 ~ 1.477)) also has positive impact on the establishment of residents’ health records. Participation in BMIUE has no impact on health education (95% CI:0.968 ~ 1.031), but has a positive impact on the establishment of health records (95%CI: 1.150 ~ 1.219). See Table 1 for details.

**Table 1:** Multivariate Logistic regression analysis of social integration of medical insurance on health education and health records

Research indicators	Health education			Health records		
	β	Exp(β)95% confidence interval		β	Exp(β)95% confidence interval	
		lower limit	upper limit		lower limit	upper limit
Age	−0.183	0.813	0.853	0.007	0.984	1.030
Gender	0.101	1.081	1.132	0.132	1.116	1.167
Marital status	0.188	1.169	1.245	0.196	1.181	1.254
Education	0.026	1.011	1.041	0.004	0.990	1.018
Household registration system	0.001	0.980	1.022	0.081	1.064	1.105
Average monthly expenditure	−0.060	0.964	0.993	−0.095	0.896	0.922
Flow range	0.197	1.198	1.236	0.248	1.263	1.300
Flow time	−0.055	0.930	0.954	−0.016	0.972	0.997
Medical insurance for urban and rural residents	0.236	1.195	1.342	0.190	1.150	1.272
BMIUE	−0.001	0.968	1.031	0.169	1.150	1.219
Political participation	0.600	1.750	1.898	0.312	1.324	1.410
Participate in the event	0.724	2.009	2.119	0.404	1.463	1.534
Identity	0.421	1.387	1.675	0.282	1.191	1.477
Constant	0.145	–	–	−1.829	–	–

### The influence of medical insurance on the use of public health services: the mediating effect of social integration

**Fig. 1:**
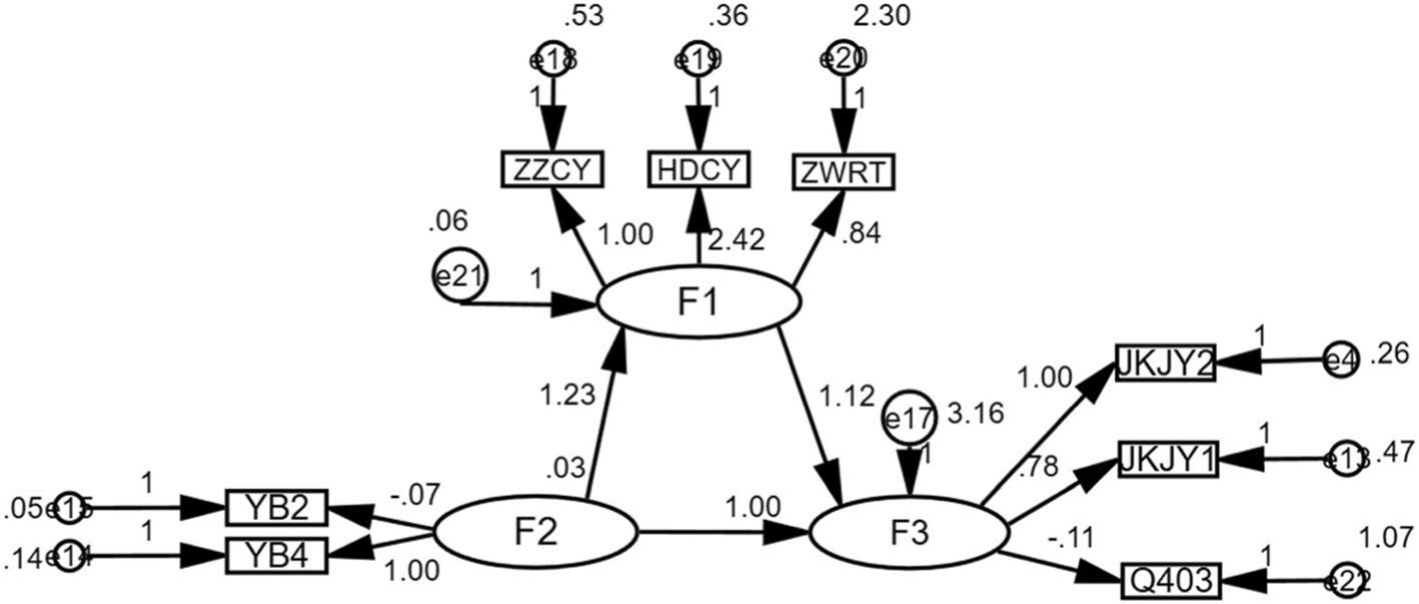
A structural equation model with social integration as an intermediary. Note: F1 on behalf of social integration;F2 on behalf of medical insurance;F3 on behalf of public health services;YB2,YB4 on behalf of urban and rural residents medical insurance, basic medical insurance for urban employees; ZZCY,HDCY,ZWRT on behalf of political participation, activity participation, identity; JKJY1,JKJY2 represents infectious diseases health education, chronic diseases health education; Q403 represents resident health records

The study took medical insurance as an independent variable, social integration as a mediating variable, and public health service utilization as a dependent variable. AMOS 25.0 was used to construct structural equation model to analyze the mediating effect of social integration. The fitting index of the model were χ^2^/*df* = 259.245, GFI = 0.993, AGFI = 0.985, CFI = 0.982, RMSEA = 0.041. The fitting index of the model was in a reasonable range and the fitting effect of the model was good. See Fig. 1 for details.

The Bootstrap method was used to analyze the mediating effect of social integration. The original data were sampled repeatedly for 1000 times and the 95% confidence interval was calculated. If the 95% CI of the normalized path coefficient did not contain 0, the mediating effect was significant. The 95% CI of the total effect of medical insurance on the utilization of public health services is 0.029–0.221, the 95% CI of the direct effect is 0.093 ~ 0.103, the 95% CI of the indirect effect is 0.108 ~ 0.127, and the total effect is direct effect. Indirect effects were significant, indicating that social integration was a partial mediator, and the mediating effect size was 57.73% (0.127/0.220). See Table 2 for details.

**Table 2:** Analysis of the results of the intermediary effect of social integration

Variable	Point estimate	Standard error	Z-value	Bootstrap Percentile 95% confidence interval			
					Lower limit		Upper limit
Total effect							
Medical insurance -Public health services	0.220	0.004	55.000		0.029		0.221
Direct effect							
Medical insurance -Public health services	0.092	0.003	30.667		0.093		0.103
Indirect effects							
Medical insurance -Public health services	0.127	0.006	21.167		0.108		0.127

## Discussion

### Medical insurance for urban and rural residents and BMIUE has an impact on the utilization of public health services

Basic public health services are a long-term and fundamental institutional arrangement in China’s medical and health sector [12]. As for the closest policies, in 2016, the CPC Central Committee and The State Council issued the outline of “Healthy China 2030”. The report of the 19th CPC National Congress emphasized the need to accelerate the equalization of basic public services and establish a high-quality and efficient medical and health service system. All policies emphasized the importance of public health services to the floating population. Meanwhile, with the outbreak of COVID-19 in the past 2 years, the prevention and treatment of infectious diseases among floating population has become more and more important. Medical insurance for urban and rural residents and BMIUE are China’s basic social medical insurance [13], and the insured object is all urban and rural residents and workers. Under the strategy of Healthy China, the linkage of medical insurance for urban and rural residents and contracted family doctor services has increased the opportunities for the insured to receive health education, improved the records of residents’ health records [14], and effectively improved the utilization of grass-roots public health services. From this point of view, the participation of floating population in medical insurance for urban and rural residents and medical insurance for urban workers is of great significance to the utilization of floating population’s public health services.

The floating population with medical insurance has a positive impact on the acceptance of health education and the establishment of health records, which is consistent with previous studies [15]. In addition, previous studies have shown that floating population with medical insurance may receive more types of health education. Single factor analysis results showed that the participation of floating population in BMIUE had positive effects on health education and health record establishment, this is consistent with the existing research [16], town worker is mutual promotion between health care and public health services utilization, after receiving health education, floating population health consciousness enhancement, will take the initiative to understand health care related policies, more willing to join the medical insurance for urban employees, Increase health protection [17]. On the contrary, participating in the urban workers’ medical insurance can reduce the economic burden of the floating population, and promote the floating population to pay attention to their own health and enjoy public health service projects. However, in the multi-factor analysis, BMIUE has no effect on the floating population’s acceptance of health education, which may be because urban workers are mostly young, in good health, have few visits to the doctor and ignore the preventive effect of health education [18]. In addition, it may also be because BMIUE has a higher reimbursement ratio, which reduces the financial burden for the floating population, so they do not attach much importance to the free health education promotion.

### Social integration acts as an intermediary between medical insurance and the utilization of public health services

The results of mediating effect analysis show that the participation in medical insurance can not only directly promote the floating population’s use of public health services, but also promote the improvement of the level of use through social integration. Basic medical insurance for urban and rural residents is a combination of the new rural cooperative medical insurance and basic medical insurance for urban residents after the Opinions of The State Council on Integrating The Medical Insurance System for Urban and Rural Residents (No.3 of The State Council [2016]) was issued in 2016. Basic medical insurance for urban and rural residents can significantly increase the social integration of floating population in the local level, this is because the integration of basic medical insurance for urban and rural residents to enjoy better medical treatment of the floating population, at the same time enhanced portability, not because of the floating population employment flow will not be able to enjoy urban medical services, especially from the rural floating population [19]. Town worker medical insurance is by the unit and individual is collective pay, personal percentage unit pay a little higher, this is actually business units on the body of this part of the employees of the floating population is the most basic health care, body condition, once the employees can submit an expense account partial medical costs, alleviate their economic burden, reduce trouble back at home, and stability in the local participate in work and life. This gives them opportunities to discuss national and social events, make suggestions for the government, participate in donation, blood donation, party and league activities, etc., enhance their identity and better integrate into the local community [20]. Moreover, social integration and equalization of access to public health services complement each other. In order to promote the integration of the floating population into local life as soon as possible, the government will formulate a series of policies to protect the rights of the floating population in local life. With more opportunities for floating population to participate in various activities, their sense of identity will also be enhanced. They will take the initiative to communicate with local residents, understand medical and health policies, and take the initiative to receive health education, regular physical examination and check health records in order to pay attention to physical health [21, 22]. Therefore, social integration plays a partially mediating role in the participation of medical insurance for urban and rural residents, medical insurance for urban workers and the utilization of public health services.

In short, the article argues that medical insurance can promote the floating population to actively accept health education and establish health records, and can further improve the utilization rate of basic public health services by improving social integration. As for the three key variables of medical insurance, social integration and utilization of basic public health services, most existing studies focus on the relationship between two variables [4–7]. On this basis, this paper further clarifies the relationship between the three variables, so as to provide a realistic basis for improving the level of utilization of basic public health services for the floating population. This is the important conclusion of this paper.

However, this study also has some limitations. The results of this study are based on mining of existing data, due to the limited variables in the original data, the reliability of some indicators is low. If one can add new effective variables in the future, the explanation of the influence of medical insurance and social integration on the utilization of basic public health services of the floating population will be more complete. In addition, a cross-sectional design was used. Because it was a self-reported measure, the study also had potential recall bias.

## Conclusions

Medical insurance can promote the floating population to actively accept health education and establish health records, and can further improve the utilization rate of basic public health services by improving social integration. Therefore, relevant government departments should promote the floating population to actively participate in local insurance, pay attention to the mediating effect of social integration, and enhance their identity. The improvement of the utilization rate of basic public health services for floating population is of profound significance for the prevention and control of chronic diseases and infectious diseases in China.

## Data Availability

The datasets generated and analysed during the current study are available in the Migrant Population Service Center, National Health Commission P.R. China repository, http://www.chinaldrk.org.cn/wjw/#/data/classify/population/yearList.
